# Retracted: Effect of Rat Medicated Serum Containing Zuo Gui Wan and/or You Gui Wan on the Differentiation of Stem Cells Derived from Human First Trimester Umbilical Cord into Oocyte-Like Cells *In Vitro*

**DOI:** 10.1155/2020/2938051

**Published:** 2020-09-30

**Authors:** Evidence-Based Complementary and Alternative Medicine

*Evidence-Based Complementary and Alternative Medicine* has retracted the article titled “Effect of Rat Medicated Serum Containing Zuo Gui Wan and/or You Gui Wan on the Differentiation of Stem Cells Derived from Human First Trimester Umbilical Cord into Oocyte-Like Cells *In Vitro*” [1], due to indications of image manipulation and duplication as raised on PubPeer [2].

The western blots in Figure 5 show signs of duplication and other manipulation. In Figure 2, the 0 day panel in (a) was duplicated in the 0 day panel in (b).

The authors provided a corrected image for the 0 day panel for 2(b) and provided high-resolution images showing no duplication in Figure 3(b) in the DAPI panels (see the supplementary materials). However, the original images for the western blots in Figure 5 were not available.

## Supplementary Materials

Supplementary Materials Corrected image for the 0 day panel for 2(b) and provided high-resolution images showing no duplication in Figure 3(b) in the DAPI panels.
