# Supplementary material for: Retracted: Effect of Rat Medicated Serum Containing Zuo Gui Wan and/or You Gui Wan on the Differentiation of Stem Cells Derived from Human First Trimester Umbilical Cord into Oocyte-Like Cells In Vitro
Source: Evid Based Complement Alternat Med. 2020 Sep 30;2020:2938051. doi: 10.1155/2020/2938051 (PMC7648685; doi:10.1155/2020/2938051)

**Figure 2(b)**

Corrected image for the 0 day panel for 2(b)


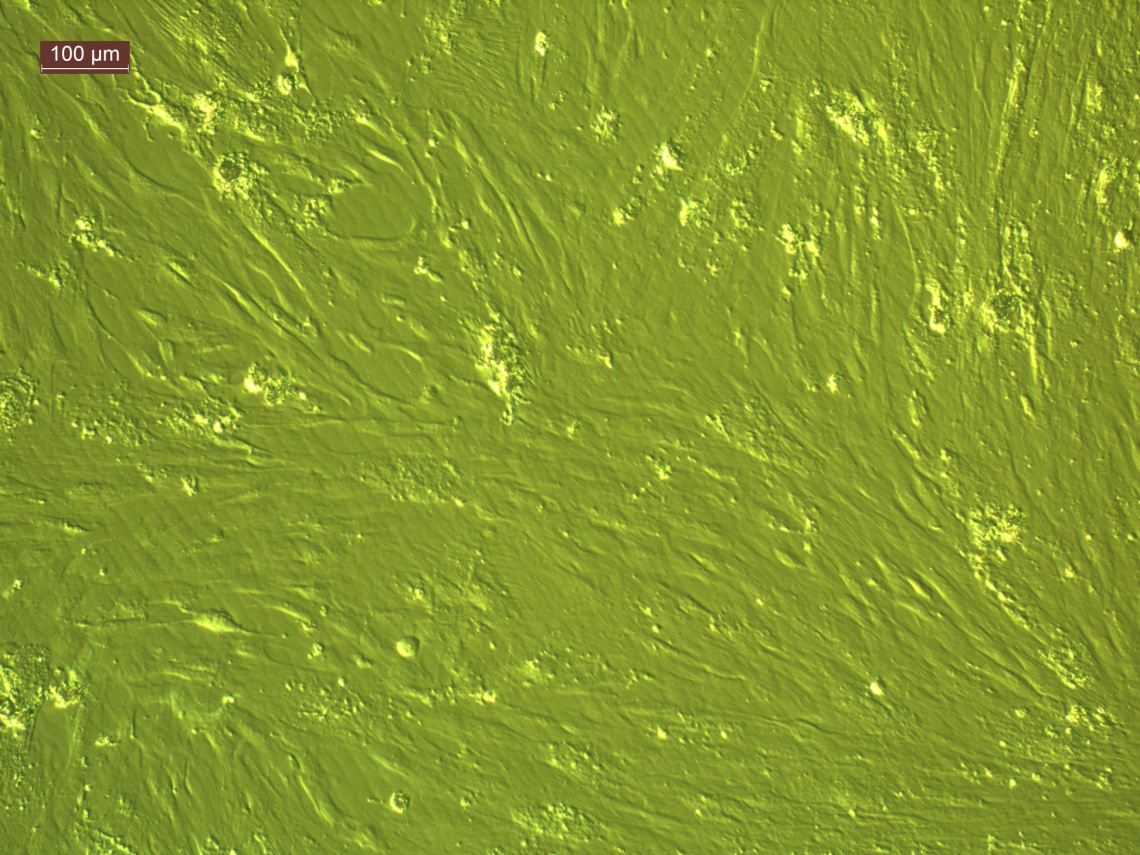


**Figure 3(b)**

High-resolution images showing no duplication in Figure 3(b) in the DAPI panels


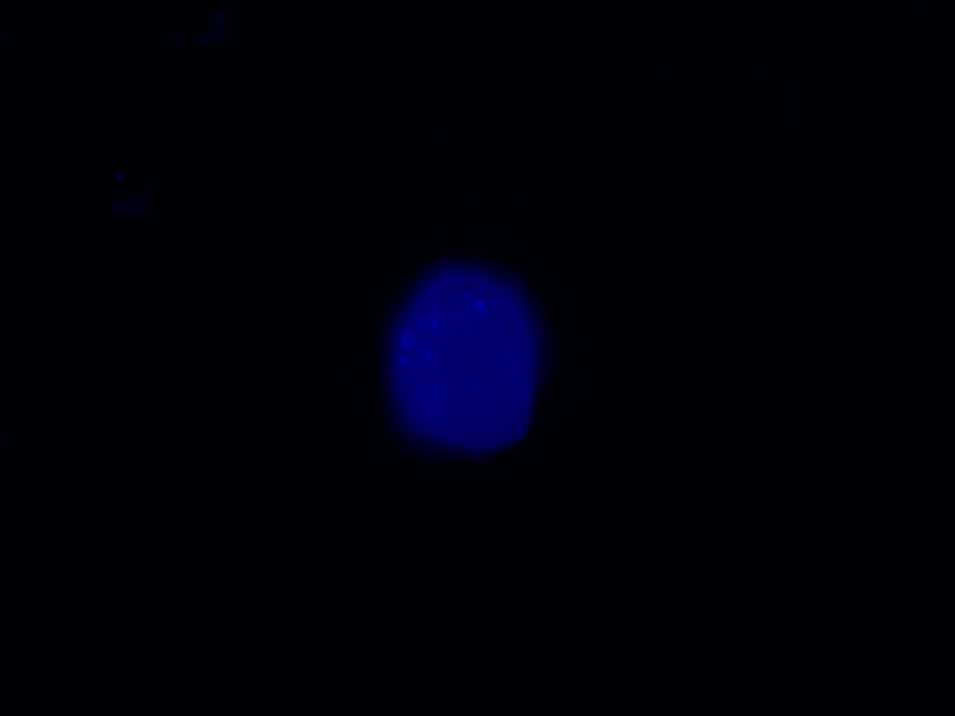

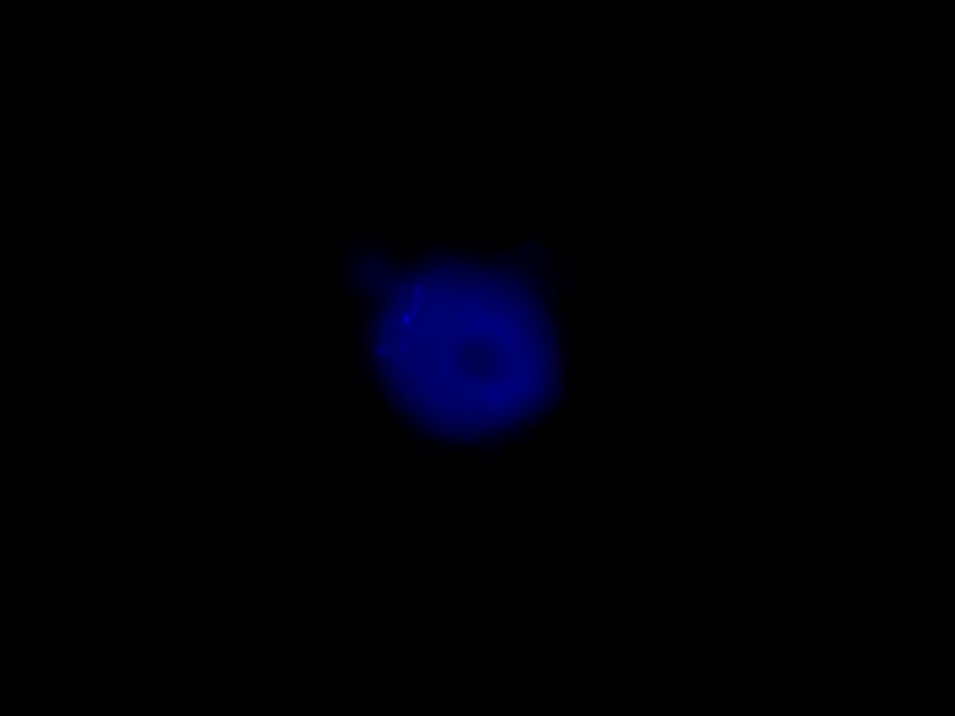

Supplement: Supplementary Materials — Corrected image for the 0 day panel for 2(b) and provided high-resolution images showing no duplication in Figure 3(b) in the DAPI panels. [file 2938051.f1.docx]
